# Coronal axis deviations in medial unicompartmental knee arthroplasty failures: an imaging study of patients revised for aseptic loosening

**DOI:** 10.1186/s40001-025-03112-2

**Published:** 2025-09-02

**Authors:** Filippo Migliorini, Nicola Maffulli, Daniel Kämmer, Ulf Krister Hofmann, Jörg Eschweiler, Andreas Bell

**Affiliations:** 1 Department of Trauma and Reconstructive Surgery, University Hospital of Halle, Martin-Luther University Halle-Wittenberg, Ernst-Grube-Street 40, 06097 Halle (Saale), Germany; 2 Department of Orthopaedic and Trauma Surgery, Academic Hospital of Bolzano (SABES-ASDAA), Via Lorenz Böhler 5, 39100 Bolzano, Italy; 3 Department of Life Sciences, Health, and Health Professions, Link Campus University, Via del Casale di San Pio V, 00165 Rome, Italy; 4 Department of Orthopaedic and Trauma Surgery, Eifelklinik St. Brigida, 52152 Simmerath, Germany; 5 Faculty of Medicine and Psychology, University “La Sapienza” of Rome, Rome, Italy; 6 School of Pharmacy and Bioengineering, Keele University Faculty of Medicine, Stoke On Trent, ST4 7QB UK; 7 Centre for Sports and Exercise Medicine, Barts and the London School of Medicine and Dentistry, Mile End Hospital, Queen Mary University of London, London, E1 4DG UK; 8 Department of Orthopaedic, Trauma, and Reconstructive Surgery, RWTH University Hospital, 52074 Aachen, Germany

**Keywords:** Lower limb alignment, Aseptic loosening, Joint line convergence angle, Anatomical–mechanical angle, Coronal malalignment, Fixed-bearing implant, Unicompartmental knee failure

## Abstract

**Purpose:**

Aseptic loosening remains a leading cause of revision in medial unicompartmental knee arthroplasty (UKA). This imaging study aimed to identify recurrent patterns of coronal alignment deviation in patients undergoing revision to total knee arthroplasty (TKA) to explore whether subtle malalignment may contribute to biomechanical failure.

**Methods:**

Imaging of patients who underwent revision surgery of a medial UKA to TKA for aseptic loosening of the tibial or femoral component was retrieved. Lower limb axes were evaluated using anteroposterior plain radiographs of the leg using the software MediCAD Knie 2D (mediCAD Hectec GmbH, Altdorf, Germany). The radiographic axes of revised patients were compared with established reference values, as defined by the MediCAD Knie 2D software and published literature, to identify common alignment patterns potentially associated with aseptic loosening.

**Results:**

Data from 62 patients were analysed. Before the revision surgery, the joint line convergence angle (JLCA, *P* = 0.002) and the anatomical–mechanical angle (AMA, *P* < 0.0001) were statistically significantly greater than the corresponding reference values. In contrast, the mechanical lateral distal femoral angle (mLDFA, *P* < 0.0001), the mechanical and anatomical medial proximal tibial angle (mMPTA and aMPTA, *P* < 0.0001), and the mechanical and anatomical lateral distal tibial angle (mLDTA and aLDTA, *P* < 0.0001) were significantly lower than reference. No statistically significant difference was found in the mechanical lateral proximal femoral angle (mLPFA, *P* = 0.9) or in the mechanical axis deviation (MAD, *P* = 0.5) when compared to normative data.

**Conclusion:**

Our cohort of patients revised from medial UKA to TKA for aseptic loosening frequently exhibited consistent deviations in lower limb alignment, particularly increased AMA and JLCA, and reduced mLDFA, mMPTA, and mLDTA. These subtle but recurrent patterns may alter load distribution across the medial compartment, contributing to implant micromotion and loosening. A detailed preoperative axis assessment may help identify patients at a higher biomechanical risk.

## Introduction

Knee osteoarthritis is common. Approximately one-third of patients develop selective osteoarthritis of the medial compartment [1, 2]. In these patients, a unicompartmental knee arthroplasty (UKA) could be indicated. The indications for a UKA were first outlined in 1989 by Kozinn and Scott [3]: mono-condylar osteoarthritis, a range of motion of at least 90°, body mass index (BMI) below 30 kg/m^2^, absence of lower limb axial deformity and ligament insufficiency. Today, these indications have become less well-defined, as contemporary practice increasingly includes patients who fall outside those classical criteria, reflecting a broader and more heterogeneous clinical application of UKA [4]. Though approximately 30–50% of all osteoarthritis knees fulfil the indication to receive a UKA, the use of these implants remains low at 2–10% [5, 6]. Previous studies confirmed that UKA leads to greater functional scores, faster recovery, and a higher satisfaction rate compared to total knee arthroplasty (TKA) [7–9]. However, UKA was associated with a greater grade of revision to TKA [9, 10]. UKA revision rates are more frequent in lower volume surgeons and institutions [11]. The reason behind this greater rate of revision is multifactorial: persistent knee pain, osteoarthritis progression of the contralateral compartment, and aseptic loosening [12]. Several clinical studies investigated the causes of the revision of a UKA to TKA. However, whether the axes of the lower limb are risk factors for aseptic loosening is still debated. A deeper understanding of the reasons behind the failure of primary UKA can help to improve patient selection and surgical indication, improving surgical outcomes.

Although numerous factors have been implicated in the failure of medial UKA, the potential contribution of subtle coronal malalignment remains underexplored. UKA relies on accurate load transfer through a limited surface area, making it particularly sensitive to deviations in femorotibial alignment. However, preoperative planning often focuses on global alignment metrics such as the mechanical axis, while overlooking angular relationships that may affect joint line orientation and compartmental stress, such as the mechanical lateral distal femoral angle (mLDFA), mechanical lateral proximal femoral angle (mLPFA), mechanical/anatomical lateral distal tibial angle (m/aLDTA), mechanical/anatomical medial proximal tibial angle (m/aMPTA), mechanical tibiofemoral angle (mTFA), anatomical-mechanical-angle (AMA), and mechanical axis deviation. A better understanding of these radiographic features in patients who undergo revision may improve surgical indications and help refine patient selection. The purpose of the present study was to evaluate, through standardised anteroposterior radiographic imaging, whether patients revised from medial UKA to TKA for aseptic loosening exhibit consistent deviations in lower limb coronal axes and angles compared to physiological reference values.

## Methods

### Study protocol

The present study was conducted in accordance with the Strengthening the Reporting of Observational Studies in Epidemiology (STROBE) guidelines [13]. This observational study was conducted in the Department of Orthopaedic and Trauma Surgery at Eifelklinik St. Brigida, Germany, and at the Department of Orthopaedics, Trauma, and Reconstructive Surgery at RWTH University Hospital Aachen, Germany. The present study was approved and registered by the Ethics Committee of RWTH Aachen University (project ID: EK128/19) and conducted in accordance with the principles outlined in the Declaration of Helsinki.

### Study setup

**Fig. 1:**
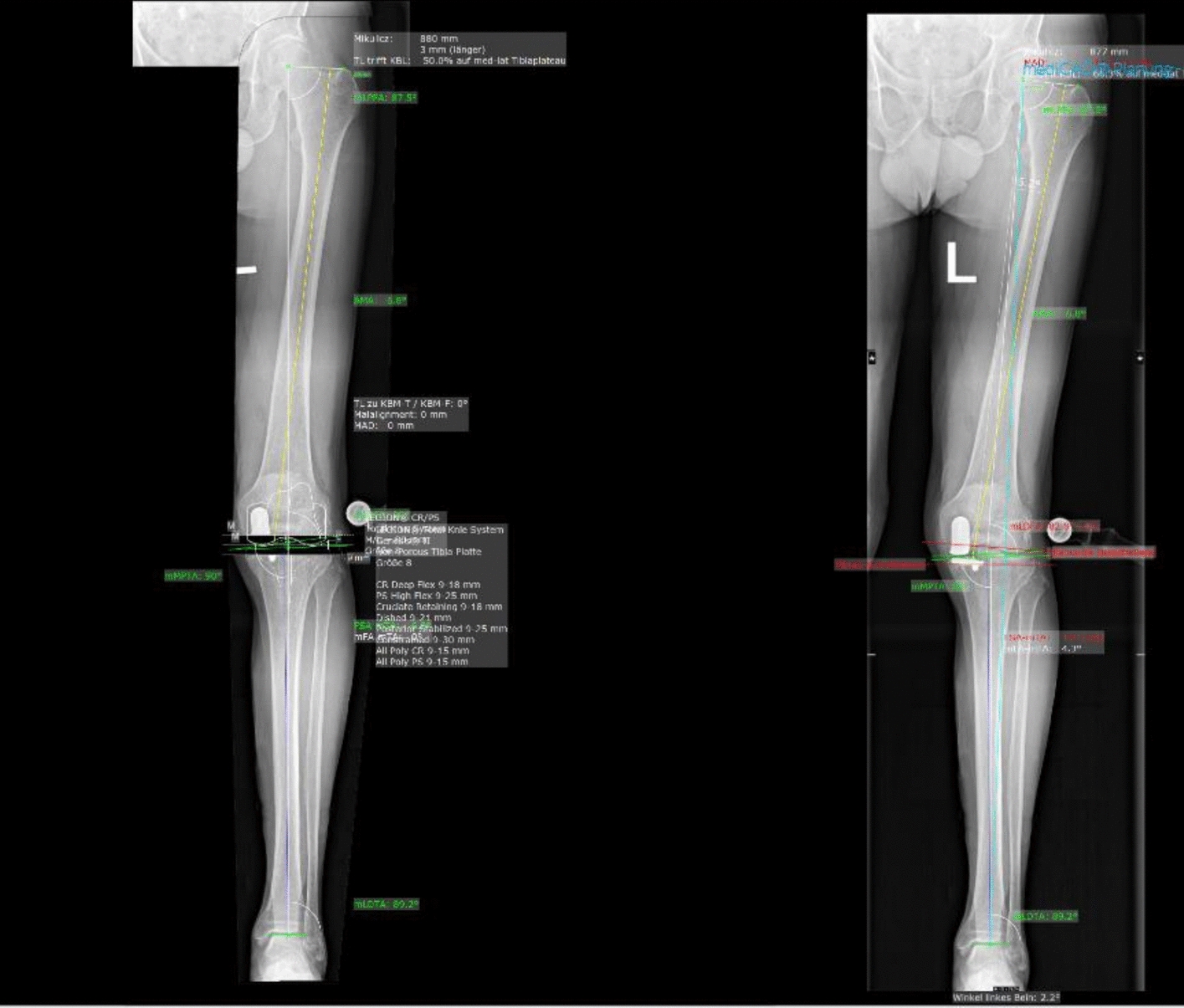
Pre-operative digital planning using MediCAD Knie 2D

Imaging data were retrieved from patients who underwent revision surgery from medial unicompartmental knee arthroplasty (UKA) to total knee arthroplasty (TKA) for aseptic loosening of the tibial or femoral component between 2016 and 2023. Coronal lower limb alignment was evaluated on standardised anteroposterior long-leg radiographs using the MediCAD Knee 2D software (mediCAD Hectec GmbH, Altdorf, Germany). This medical templating platform provides digital tools for pre- and post-operative planning in hip, knee, shoulder, trauma, and spine surgery, and includes integrated algorithms for the automated measurement of lower limb axes. In the present study, the radiographic parameters provided by the MediCAD Knee 2D software (Fig. 1) were collected for each patient and compared to the corresponding reference values. These reference values are derived from the software’s internal normative database, which is based on healthy adults with neutral mechanical alignment, and are consistent with those reported in previously published anatomical studies [14–18]. All measurements were obtained from radiographs acquired after medial UKA implantation but prior to revision surgery. These images were used for preoperative planning of the revision procedure and reflect the coronal alignment of the limb at the time of prosthesis failure.

### Eligibility criteria

**Fig. 2:**
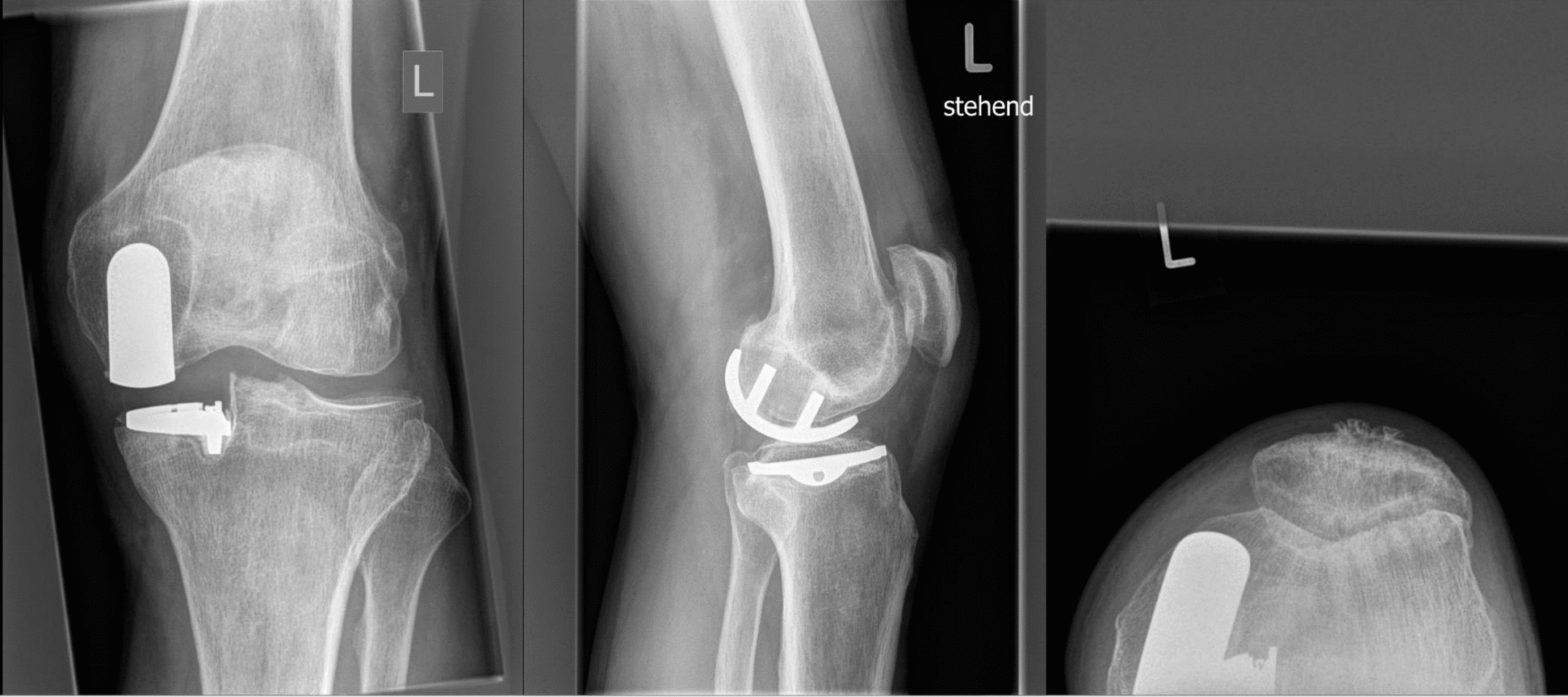
Plain radiographs of a 75-year-old female patient with aseptic loosening of the tibial component

The inclusion criteria were: (1) patients who underwent TKA as revision surgery for failed medial cemented UKA for unicondylar knee OA; (2) only patients who received fixed-bearing cemented implants were considered; (3) patients with evidence of aseptic loosening of the tibial and/or femoral component at imaging (Fig. 2); (4) evidence of non-infected samples during the preoperative joint punctate and the intraoperative microbiologic examination; (5) patients being able to understand the nature of the treatment. The imaging examination for the diagnosis of aseptic loosening included plain radiographs, computer tomography (CT), and single-photon emission computed tomography (SPECT/CT).

The exclusion criteria were: (1) patients who underwent revision surgery of UKA to TKA for other aetiologies rather than aseptic loosening (e.g. trauma, infections, neoplasia); (2) presence of bone defect greater than 5 mm in its longer axis; (3) chronic or acute inflammatory diseases; (4) neoplastic diseases; (5) pregnancy; (6) any blood abnormalities; (7) immunodeficiency; (8) severe peripheral neuropathy, vascular diseases, or presence of peripheral ulcers; (9) other omitted criteria which may have influenced the results of the present investigation. Only patients with confirmed aseptic loosening of the medial unicompartmental knee arthroplasty were included. Final diagnosis of loosening was established intraoperatively, based on direct observation of macromotion of the femoral or tibial component during revision surgery. Cases with suspected radiographic loosening but intraoperative evidence of stable fixation were excluded.

### Surgical revision of UKA

All patients who underwent revision procedure received preoperative intravenous antibiotic prophylaxis with 1.5 g cefuroxime. The surgeries were performed in a supine position on a radiotransparent operating table with a tourniquet at the thigh 150 mmHg over the patient’s systolic arterial pressure. A medial parapatellar approach was performed as standard. All patients received a posterior stabilised Legion Revision Knee System modular revision prosthesis, condylar constrained inlay, with CoCr intramedullary tibial stem (Smith&Nephew plc., London, UK). All implants were cemented using gentamicin-loaded bone cement PALACOS + G (Heraeus Kulzer GmbH, Hanau, Germany). A ReX cement stop TM (Spierings Orthopaedics BV, Nijmegen, Netherlands) was used to avoid intramedullary cement migration. Four microbiological swabs were collected intraoperatively. One gram of tranexamic acid diluted into 50 ml NaCl was injected into the joint after wound closure. Closed suction intra-articular and subcutaneous drains were used for 48 h. On the fifth postoperative day, all patients underwent lateral and anteroposterior radiographs of the operated leg.

### Data assessment

All data were prospectively collected by one physician involved in the clinical management of the patients. All personal patient data were retrieved at admission. Data of patients were provided to two assessors, who were blinded to personal data and not involved in the clinical management of patients. Age at surgery, women, right side, the time elapsed from UKA to TKA, and the length of hospitalisation were analysed. The preoperative digital planning was performed on admission using anteroposterior radiographs of the leg.

**Fig. 3:**
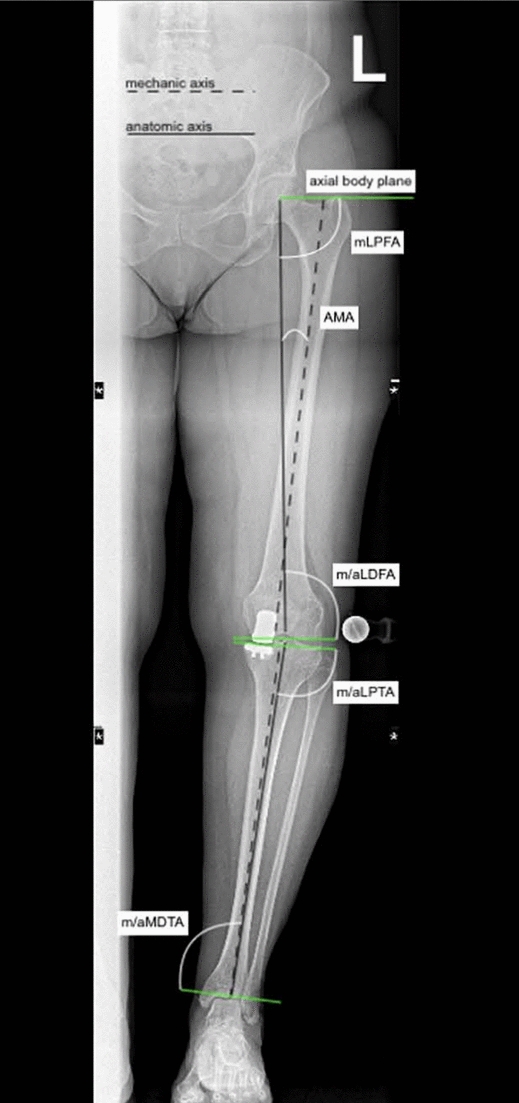
Reference values for coronal alignment parameters based on the MediCAD Knee 2D software (mLDFA: mechanical lateral distal femoral angle; mLPFA: mechanical lateral proximal femoral angle; m/aLDTA: mechanical/anatomical lateral distal tibial angle; m/aMPTA: mechanical/anatomical medial proximal tibial angle; mTFA: mechanical tibiofemoral angle; AMA: anatomical–mechanical angle; MAD: mechanical axis deviation)

The mechanical leg axis runs as a connecting line between the centre of the femoral head and the centre of the ankle joint. Physiologically, the mechanical leg axis runs medially to the centre of the knee joint. The anatomical axes of the femur and tibia correspond to the median lines of the long bones. In physiological conditions, they form a laterally open angle. The mechanical axis of the femur is defined as the connecting line between the centre of the femoral head and the knee, and the mechanical axis of the tibia as the connecting line between the centre of the knee joint and the ankle joint. In the femur, the anatomical and mechanical axes form an angle, namely the anatomical–mechanical angle (AMA). The anatomical and mechanical axes of the tibia are similar. The knee baseline (tangent to the femoral condyles) and the tibial plateau line form the joint line convergence angle (JLCA). Physiologically, the two tangents run approximately parallel. The anatomical and mechanical joint angles of the knee and ankle are determined by the angle between the base of the knee and the line of the ankle and the respective axes. In physiological conditions, the mechanical lateral distal femoral angle (mLDFA) is 87° ± 3°. To the knee baseline, the anatomical femoral axis forms a distal lateral femoral angle (aLDFA). The large range of motion, especially in flexion, is due to the anatomy of the femoral and tibial diaphysis and the configuration of the articular condyles. In the sagittal plane, both the femoral and tibial diaphysis are concavely curved dorsally. The tibial plateau is inclined dorsally (tibial slope) and offset slightly dorsally compared to the axis of the femoral shaft. The most relevant lower limb axes and angles are reported in Fig. 3. An explanation of the imaging references used is shown in Table 1.

**Table 1:** Imaging parameters

Abbreviation	Definition	Mean (°)	Range (°)	Description
mLDFA	Mechanical lateral distal femoral angle	88°	85–90	The angle between the lateral femur and the femoral joint
mLPFA	Mechanical lateral proximal femoral angle	90°	85–95	The angle between the mechanical femoral shaft axis and the orientation line through the centre of the femoral head and the tip of the greater trochanter
m/aLDTA	Mechanical/anatomical lateral distal tibial angle	89°	86–92	The angle between the mechanical axis of the tibia and the tibial plateaux, measured on the lateral side
m/aMPTA	Mechanical/anatomical Medial proximal tibial angle	87°	85–90	The angle between the mechanical axis of the tibia and the tibial plateau knee joint line, measured on the medial side
JLCA	Joint line convergence angle	2° medial	0–3	Interaction of the intra-articular deformity arising from the osteoarthritis and the surrounding soft tissue laxity
MAD	Mechanical axis deviation	10 mm medial	3–17	Distance from the centre of the knee joint and the line of the mechanical axis
mTFA	Mechanical–tibiofemoral angle	1.25°	1–3.2	The angle between the mechanical axis of the femur and the mechanical axis of the tibia, also known as the hip–knee–ankle angle
AMA	Anatomical–mechanical angle	6°	5–7	The angle between anatomical and mechanical femoral axes

### Statistical analysis

The statistical analyses were performed by the main author (**). All statistical analyses were performed using the software IBM SPSS version 25. The mean values calculated for each radiographic parameter in the revision cohort were compared with the corresponding normative mean reference values using one-sample statistical testing. For representative statistics, the arithmetic mean and standard deviation were used. For the analysis of continuous data, the mean difference (MD) effect measure was used. The unpaired two-tailed *T*-test was performed, with values of *P* < 0.05 considered statistically significant.

## Results

### Recruitment process

**Fig. 4:**
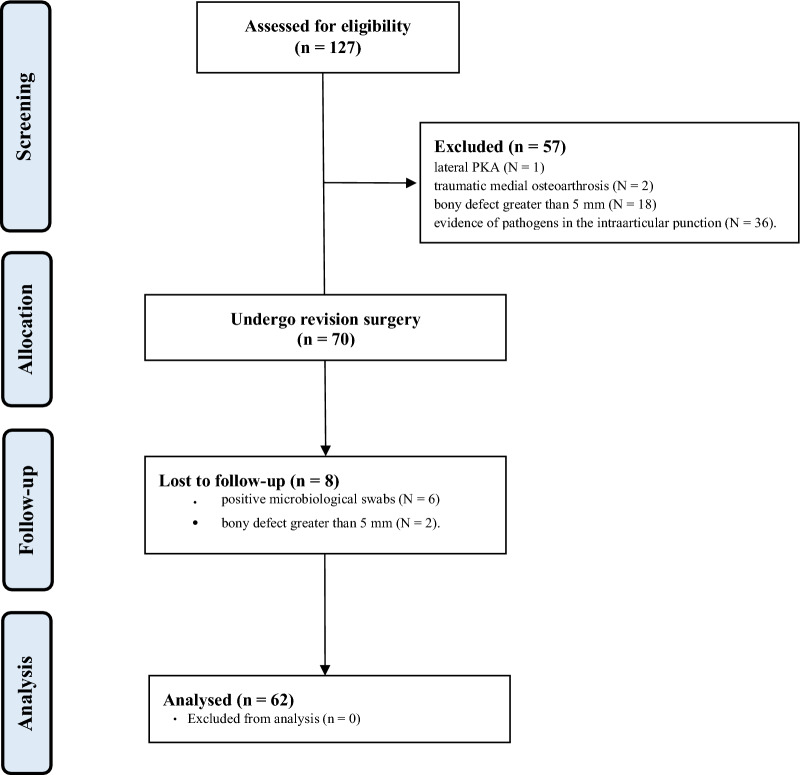
STROBE diagram of the recruitment process

Data from 127 patients were retrieved. Of them, 57 were excluded for the following reasons: lateral UKA (*N* = 1), traumatic medial osteoarthrosis (*N* = 2), bony defect greater than 5 mm (*N* = 18), evidence of pathogens in the intra-articular puncture (*N* = 36). 70 patients underwent revision surgery. A further eight patients were excluded as they intraoperatively demonstrated positive microbiological swabs (*N* = 6) or bony defects greater than 5 mm (*N* = 2). Finally, data from 62 patients were considered in the present investigation (Fig. 4).

### Patient demographics

Data from 62 patients were collected (Table 2). 55% (34 of 62 patients) were women. 45% (28 of 62 procedures) were on the right side. The mean age of the patients was 66.9 ± 10.8 years, and the mean BMI was 27.9 ± 3.1 kg/m^2^. The time elapsed from UKA to revision was 73.3 ± 59.1 months. The mean length of the hospitalisation was 8.7 ± 1.4 days, and the mean preoperative range of motion was 109.4 ± 14.8°.

**Table 2:** Analysis of mechanical and anatomical axes (mLDFA: mechanical lateral distal femoral angle; mLPFA: mechanical lateral proximal femoral angle; m/aLDTA: mechanical/anatomical lateral distal tibial angle; m/aMPTA: mechanical/anatomical medial proximal tibial angle; JLCA: joint line convergence angle; MAD: mechanical axis deviation; mTFA: mechanical tibiofemoral angle; AMA: anatomical–mechanical angle)

Values	Pre-OP	MD	P
JLCA	3.7 ± 3.9	1.7	0.002
mLPFA	90.1 ± 6.6	0.1	0.9
mLDFA	86.5 ± 3.0	− 1.5	0.0002
mMPTA	83.2 ± 6.2	− 3.8	< 0.0001
mLDTA	85.1 ± 3.8	− 3.9	< 0.0001
AMA	7.2 ± 1.1	1.2	< 0.0001
MAD	11.9 ± 21.2	1.9	0.5

Reference values used for comparison are reported in Table 1

### Results syntheses

The preoperative JLCA (*P* = 0.002) and AMA (*P* < 0.0001) were greater than the reference value. The preoperative mLDFA (*P* < 0.0001), mMPTA (*P* < 0.0001), and mLDTA (*P* < 0.0001) were lower than the reference value. The most frequently altered parameter was AMA, abnormal in 61.3% (*N* = 38), followed by mMPTA in 58.1% (*N* = 36), mLDTA in 48.4% (*N* = 30), MAD in 41.9% (*N* = 26), and JLCA in 38.7% (*N* = 24) of cases. No difference was found in mLPFA (*P* = 0.9) and MAD (*P* = 0.5). These results are shown in Table 2.

## Discussion

The optimal alignment of the lower limb following arthroplasty remains a topic of debate. The present study investigated the coronal alignment patterns in patients who underwent revision from medial UKA to TKA due to aseptic loosening. The analysis revealed consistent deviations in specific radiographic parameters when compared to reference values. Statistically significant increases were observed in JLCA and AMA, while mLDFA, mMPTA and mLDTA were significantly reduced. These findings suggest that, despite the absence of gross frontal malalignment as indicated by a non-significant difference in MAD, the internal architecture and joint line orientation of the limb showed subtle but relevant deviations. The AMA was significantly greater than the reference value, and this was the most frequently altered parameter, being abnormal in 61.3% (*N* = 38) of patients. An increased AMA indicates a greater divergence between the anatomical and mechanical axes of the femur, potentially leading to increased medial loading and shear forces at the cement–bone interface. This mechanical discrepancy may predispose to micromotion and failure of implant fixation over time, particularly in fixed-bearing constructs with limited tolerance for malalignment. Similarly, the mMPTA was reduced in 58.1% (*N* = 36) of patients, indicating a varus inclination of the tibial joint surface. This configuration shifts the load-bearing axis medially, increasing compressive forces on the medial tibial plateau and possibly contributing to cement fatigue or bone resorption. The mLDTA was also reduced in nearly half of the cohort, suggesting a systematic alteration of the distal tibial alignment that may interact with the load transfer during gait. Although the MAD was not statistically different from normative values, it was abnormal in 41.9% (*N* = 26) of patients, further supporting the presence of clinically relevant malalignment despite group-level averages. The JLCA, altered in 38.7% (*N* = 24), reflects intra-articular deformity and soft tissue laxity, and its elevation suggests asymmetric joint line convergence and medial compartment overload. Notably, 92% of patients presented with at least two parameters outside physiological ranges, with the most common combination involving AMA, mMPTA, and mLDTA. This finding highlights the presence of a recurrent biomechanical phenotype among patients undergoing revision, supporting the hypothesis that subtle coronal malalignment, even in the absence of gross deformity, may contribute to aseptic loosening in medial UKA.

**Fig. 5:**
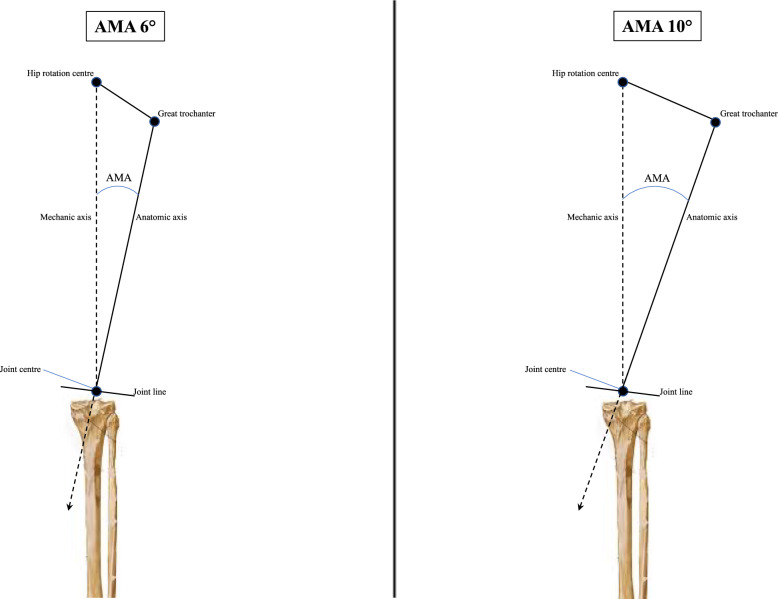
Schematic representation of the biomechanics of the lower limb with a 6° and 10° AMA

Alterations in coronal alignment, even within so-called “acceptable” limits, may exert substantial biomechanical effects on medial UKA. An increased AMA amplifies the divergence between the anatomical and mechanical femoral axes, shifting the functional load-bearing vector medially (Fig. 5). This altered vector increases the compressive and shear stresses on the medial tibial plateau, concentrating forces at the cement–bone or implant–bone interface. A reduced mMPTA compounds this effect by inclining the tibial joint surface into varus, further medialising the mechanical axis and exacerbating compartmental overload. Unlike TKA, where the implant spans the entire joint and distributes load more evenly, a medial UKA bears concentrated stress across a much smaller surface, rendering it more sensitive to axis deviations. Moreover, a reduced mLDFA may indicate subtle femoral malposition, which reduces congruity and increases the risk of edge loading. The combination of these angles leads to a biomechanical environment characterised by increased joint line obliquity, asymmetric load transfer, and high interface pressure on the medial side. These conditions may predispose to micromotion, cement fatigue, and progressive osteolysis, ultimately resulting in aseptic loosening. The additive role of elevated JLCA, reflecting intra-articular deformity and soft tissue imbalance, further disrupts load symmetry and may contribute to implant instability. Therefore, these axis alterations, though sometimes overlooked, represent a biomechanical continuum of risk that may compromise implant longevity in medial UKA.

The lower limb angles and axes of each patient included in the present study were evaluated using MediCAD Knie 2D software and compared to their respective reference values. The reference values were obtained from the MediCAD Knie 2D software and previously published reports [14–18]. Only medial UKA were investigated in the present study. Given the differences in kinematics, load distribution, and patterns of cartilage wear, medial and lateral UKAs have different failure modalities [19, 20]. Moreover, according to national registries, up to 95% of UKAs are of the medial compartment [21–23]. Rotational errors in radiographs can also lead to an over- or underestimation of the axis of the lower limb, thereby negatively impacting the reliability of our conclusions. The reference values were obtained from the MediCAD Knie 2D software and previously published reports [14–18]. By definition, these reference values are subject to biodiversity, and considering their small size, the generalisability of our results is limited. Only patients who received fixed-bearing cemented implants were considered in the present study. Mobile-bearing UKAs, given their larger contact area and congruent bearing surfaces, are supposed to minimise constraints and contact stress, and reproduce better anatomic knee motion, thereby reducing inlay wear [24–27]. On the other hand, mobile-bearing UKAs are very sensitive to soft tissue balancing, and inlay dislocation is more common than in fixed-bearing implants [28, 29]. Fixed-bearing UKAs are more commonly used and easier to implant [28]. However, the flat tibial component of fixed-bearing UKAs, given their fatigue and shear stress-related mechanism, is less compliant during flexion and can lead to point loading, increasing the risk of inlay surface deformation and delamination [30, 31]. Only cemented UKAs were considered in the present study. Most UKAs are fixed to the bone using bone cement (e.g. polymethylmethacrylate, PMMA); however, more recently, cementless porous-coated UKAs have been introduced [32]. Inadequate cementing technique could increase stress shielding due to high loads on small surface areas and within a small operative area [33, 34]. In a recent registry study on 8733 UKAs using the Oxford Unicompartmental Knee, the risk of revision for aseptic loosening and pain doubled with the use of cement fixation [35]. Finally, the limited sample size represents another important limitation, negatively impacting the generalisability of our conclusions. While the inclusion of a non-revised control group might have provided comparative clinical data, the objective of this study was not to determine risk thresholds or predictors of failure, but rather to describe radiographic patterns at the time of revision. The reference values used in this study reflect standard mechanical alignment targets and serve as an appropriate normative comparator to identify deviations relevant to UKA biomechanics. While this study did not directly assess implant positioning or surgical technique at the time of the primary UKA, the overall limb alignment may still reflect the biomechanical consequences of both pre-existing deformity and surgical execution. Even UKAs performed with mechanical alignment principles may result in coronal axis deviations if soft tissue balancing is suboptimal or if the tibial and femoral resections do not compensate for constitutional varus. Subtle malalignment may go unrecognised on short-leg radiographs and contribute to increased medial loading and asymmetric stress distribution. Therefore, regardless of whether the prosthesis was anatomically or mechanically aligned, residual coronal deviations may have biomechanical relevance and influence implant survival.

## Conclusion

Our cohort of patients revised from medial UKA to TKA for aseptic loosening frequently exhibited consistent deviations in lower limb alignment, particularly increased AMA and JLCA, and reduced mLDFA, mMPTA and mLDTA. These subtle but recurrent patterns may alter load distribution across the medial compartment, contributing to implant micromotion and loosening. A detailed preoperative axis assessment may help identify patients at a higher biomechanical risk. These results should be validated on a larger scale.

## Data Availability

The datasets generated during and/or analysed during the current study are available upon reasonable request to the Corresponding Author.
